# The EphA2 Receptor Regulates Invasiveness and Drug Sensitivity in Canine and Human Osteosarcoma Cells

**DOI:** 10.3390/cells13141201

**Published:** 2024-07-16

**Authors:** Evelyn D. Harris, Jessica C. Sharpe, Timothy Strozen, Shabnam Abdi, Maya Kliewer, Malkon G. Sanchez, Natacha S. Hogan, Valerie MacDonald-Dickinson, Franco J. Vizeacoumar, Behzad M. Toosi

**Affiliations:** 1 Department of Small Animal Clinical Sciences, Western College of Veterinary Medicine, University of Saskatchewan, 52 Campus Drive, Saskatoon, SK S7N 5B4, Canada; evelyn.harris@usask.ca (E.D.H.); jessica.sharpe@usask.ca (J.C.S.); timothy.strozen@usask.ca (T.S.); shabnam.abdi@usask.ca (S.A.); maya.kliewer@usask.ca (M.K.); mas925@mail.usask.ca (M.G.S.); valerie.macdonald@usask.ca (V.M.-D.); 2 Department of Biochemistry, Microbiology and Immunology, College of Medicine, University of Saskatchewan, GA20 Health Sciences, 107 Wiggins Road, Saskatoon, SK S7N 5E5, Canada; 3 Department of Animal and Poultry Science, College of Agriculture and Bioresources, University of Saskatchewan, 51 Campus Drive, Saskatoon, SK S7N 5A8, Canada; natacha.hogan@usask.ca; 4 Cancer Research, Saskatchewan Cancer Agency and Division of Oncology, University of Saskatchewan, Health Sciences Building, 107 Wiggins Road, Saskatoon, SK S7N 5E5, Canada; franco.vizeacoumar@usask.ca

**Keywords:** comparative oncology, EphA2, osteosarcoma, cancer invasion

## Abstract

Osteosarcoma is an aggressive bone cancer affecting both humans and dogs, often leading to pulmonary metastasis. Despite surgery and chemotherapy being the primary treatment modalities, survival rates remain low in both species, underscoring the urgent need for more efficacious therapeutic options. Accumulating evidence indicates numerous biological and clinical similarities between human and canine osteosarcoma, making it an ideal choice for comparative oncological research that should benefit both species. The EphA2 receptor has been implicated in controlling invasive responses across different human malignancies, and its expression is associated with poor prognosis. In this study, we utilized a comparative approach to match EphA2 functions in human and canine osteosarcoma models. Our objectives were to assess EphA2 levels and its pro-malignant action in osteosarcoma cells of both species. We found that EphA2 is overexpressed in most of both canine and human osteosarcoma cell lines, while its silencing significantly reduced cell viability, migration, and invasion. Moreover, EphA2 silencing enhanced the sensitivity of osteosarcoma cells to cisplatin, a drug commonly used for treating this cancer. Furthermore, inhibition of EphA2 expression led to a significant reduction in tumor development capability of canine osteosarcoma cells. Our data suggest that these EphA2 effects are likely mediated through various signaling mechanisms, including the SRC, AKT, and ERK–MAPK pathways. Collectively, our findings indicate that EphA2 promotes malignant behaviors in both human and canine osteosarcoma and that targeting EphA2, either alone or in combination with chemotherapy, could offer potential benefits to osteosarcoma patients.

## 1. Introduction

Osteosarcoma (OS) is an aggressive and often metastatic bone cancer in both humans and dogs. In dogs, OS accounts for most primary bone tumors, mostly affecting large breeds [1,2]. In humans, OS affects adolescents, with the median age at diagnosis being 16 years old [3]. The current standard of care for this cancer involves both limb amputation or sparing surgery and chemotherapy [3,4]. However, despite this aggressive treatment regimen, survival rates remain low. With treatment, the average survival rate for dogs is less than one year, dropping to less than 3 months without treatment [4,5]. For humans, treatment leads to a survival rate of 60% in patients with primary tumors and no metastases; however, the 5-year survival rate decreases to 20–30% with metastatic disease [4,5].

Treatment options for OS and its clinical outcomes have remained stagnant, reflecting challenges such as limited disease understanding, lack of biomarkers for early diagnosis, and complexities in the tumor microenvironment [6,7]. Additionally, OS is considered a rare disease, resulting in minimal funding and research activity, hindering efforts to improve clinical outcomes [4,5,7]. Since many human and canine cancers, including OS, are similar in clinical presentations and biology [8], and cancers in dogs and humans often exhibit close genetic links, studying canine OS could yield novel insights into this disease in both species [8]. Given the substantially higher incidence rate in dogs (around 20:1) and their faster tumor development compared to humans, employing a comparative approach is crucial for investigating this malignancy [2]. Thus, we can study new therapeutic targets and treatment options more quickly in dogs, ultimately bringing that knowledge to OS treatment in humans.

To develop new treatment options, it is important to understand the pathobiology of the disease. Specific signaling molecules essential for cancer cells, for example, could be exploited by targeted therapies. Eph receptors form the largest group of receptor tyrosine kinases (RTKs). Overall, there are 14 different Eph receptors, including nine EphAs (EphA1–8 and A10) and five EphBs (EphB1–4 and B6) expressed in mammalian cells. These molecules, found on the cell membrane, bind to their ephrin ligands presented on neighboring cells [9]. Once active, the Eph receptors can transduce a variety of signals inside the cell, which are responsible for essential cell functions such as proliferation, differentiation, and motility [10]. Earlier studies have shown that in many malignancies, multiple Eph receptors are differentially expressed when compared to normal tissue [9,11]. In particular, EphA2 is the most common Eph receptor to be expressed irregularly in human tumors, and its increased expression has been associated with metastatic phenotypes [9]. 

Here, we aimed to investigate the action of EphA2 in human and canine OS. In the course of our work, we found an increased expression of EphA2 in both human and canine OS cells. Consistent with this, EphA2 silencing suppressed the proliferation, migration, and invasive activity of human and canine OS cells. Our data also indicate that EphA2 significantly contributes to OS cell migration/invasion and tumor growth and reduces the sensitivity of OS cells to a commonly used chemotherapeutic drug, cisplatin, potentially through the activation of SRC, AKT and/or ERK–MAPK pathways. Taken together, the results of this study suggest that EphA2 is a driver of the malignant behavior of canine and human OS and is a promising therapeutic target in these malignancies.

## 2. Materials and Methods

### 2.1. Antibodies and Reagents

Anti-EphA2 antibody (#12927), anti-Tubulin (#3873), anti-p-mTOR (#5536), anti-p-Akt (#4060), anti-N-Cadherin (#13116), anti-integrin β3 (#13166), anti-p-p44/42 MAPK (#4370), anti-p-Src (#6943), and anti-mouse IgG1 (#5415) were from Cell Signaling Technologies, Danvers, MA, USA. Anti-GAPDH (sc-47724) was from Santa Cruz Biotechnology, Dallas, TX, USA. Recombinant ephrin-A1 Fc was from R&D Systems (#6417-A1). 

### 2.2. Cell Culture 

Abrams, McKinley, and Gracie cell lines were obtained from Colorado State University. Payton, Eva, and Igler cell lines were from the University of Wisconsin—Madison. Canine osteoblast (CnOb) cells were obtained from Cell Applications Inc., San Diego, CA, USA. The following cell lines were purchased from the American Type Culture Collection (ATCC; Manassas, VA, USA): D17, U2OS, SAOS-2, SJSA1, MG63, and 143B. Human osteoblast cell line, hOb, was obtained from PromoCell. Abrams, McKinley, Gracie, D17, Payton, Igler, and Eva cells were cultured in RPMI 1640 (Hyclone, Cytiva, Wilmington, DE, USA) medium supplemented with penicillin/streptomycin (Hyclone, 100 U/mL and 100 ug/mL, respectively), MEM NEAA (Gibco, Life Technologies, Waltham, MA, USA), 1 mM sodium pyruvate (Hyclone) and 10% FBS (Gibco). All other cell lines were cultured according to the suppliers’ instructions. Cells were cultured for less than three months at a time, and Mycoplasma testing was performed. 

Canine osteoblast cells were isolated from three dogs that were euthanized at the Veterinary Medical Center at the University of Saskatchewan due to non-cancer-related reasons. The owner’s consent was obtained. Osteoblast cells were isolated according to established protocols [12,13] with minor modifications. Briefly, pieces of bone from the proximal femur were harvested under sterile conditions and transported to the laboratory in sterile phosphate-buffered saline (PBS, Hyclone) with penicillin/streptomycin. Bone pieces were aseptically cut into 1–2 mm fragments and rinsed with PBS and DMEM containing penicillin–streptomycin (3 times each, 100 U/mL and 100 µg/mL, respectively). The medium was removed, and 10 mL of 0.25% trypsin was added. Pieces were incubated at 37 °C for 10 min. Pieces were then rinsed in DMEM again and then were digested with 1:3 diluted collagenase II from Clostridium Histolyticum (MilliporeSigma, Oakville, ON, Canada) for 2 h at 37 °C with vigorous agitation every 20 min. After digestion, pieces were then rinsed with the medium and transferred to culture dishes with DMEM containing 10% FBS, MEM NEAA, and pen/strep (same concentrations as above). To allow osteoblast cells to detach from bone pieces and grow on a culture plate, these bone fragments were maintained in culture for up to 3 weeks, and the medium was changed every 2–3 days. 

### 2.3. Transduction with shRNA

To make lentiviral particles encoding EphA2-targeting shRNA or non-silencing shRNA (control), HEK 293T cells at approximately 90% confluency were co-transfected with 6 μg of plasmid encoding EphA2-targeting shRNA (Sigma) or control non-silencing shRNA, and 60 µL of packaging mix containing key HIV packaging genes and vesicular stomatitis virus G-protein envelope vector (SHP001, Sigma) in 10 mL of DMEM containing 2% FBS and 60 µL of METAFECTENE PRO (Biontex Laboratories, München, Germany). After incubating the cells for 16 h, the transfection medium was replaced with a regular culture medium. Medium, containing viral particles, was collected 48 and 72 h after transduction and filtered using 0.45 μm filters to remove cell debris. Cells were transduced with lentiviral particles in the presence of 10 μg/mL polybrene (MilliporeSigma, Oakville, ON, Canada) overnight before replacing the medium with cell culture medium and incubation for an additional 48 h. Cells were cultured in the presence of 7 µg/mL puromycin (Gibco) for 5 days for selection of cells with successful insertion of the resistance factor. Western blotting was used to confirm the silencing of the EphA2 molecule.

### 2.4. Western Blot Analysis

Once cells reached 80–90% confluency, cells were washed with cold PBS and lysed with lysis buffer [14], which contained protease and phosphatase inhibitors (Halt™ Protease and Phosphatase Inhibitor, Thermo Fisher, Waltham, MA, USA). Cells in the lysis buffer were kept on ice and vortexed every 10 min for a total of 30 min. Cells were centrifuged, and the supernatant was mixed in a 3:1 ratio with 4X Laemmli sample buffer (Bio-Rad, Mississauga, ON, Canada). Samples were heated for 6 min at 98 °C to denature. Samples were run on polyacrylamide gel (Bio-Rad) and transferred to a PVDF membrane (Bio-Rad) using a Trans-Blot Turbo Transfer System (Bio-Rad). A blocking solution of tris-buffered saline (TBS) containing 0.1% Tween-20 and 7% dry milk powder was added to the membranes and left on a rocker for 1 h. The membranes were washed with TBS and incubated in primary antibody overnight at 4 °C. Membranes were washed and kept in a matching secondary antibody diluted in TBS with 0.1% Tween 20 and 5% milk powder for 1 h in the dark. The membranes were imaged with the LI-COR Odyssey Clx imaging system (LI-COR Biosciences, Lincoln, NE, USA). Image Studio 5.2 and Carestream MI SE 5.4.2 software were used for quantification of Western blot data. Figures were generated using Image Studio and PowerPoint (Version 2404) software.

### 2.5. Immunofluorescence

Cells were grown on glass coverslips until they reached 50–60% confluency. Cells were fixed with 4% paraformaldehyde for 10 min at room temperature, rinsed with PBS and permeabilized with 0.2% Triton X-100 (Sigma–Aldrich) in PBS and incubated in blocking solution (1% BSA + 22.52 mg/mL glycine in PBST (PBS + 0.1% Tween 20)) for 30 min at room temperature on a rocker. Cells on coverslips were then incubated with a primary antibody or a matching isotype control IgG overnight at 4 °C. Cells were then incubated with a goat anti-mouse Alexa Fluor 488 secondary antibody (Invitrogen, Waltham, MA, USA) diluted in PBS with 0.1% Tween 20 and 1% BSA for 45 min at room temperature, then rhodamine phalloidin (4 µL of 40× stock solution, 1× final concentration) was added to the secondary antibody solution, and incubation continued for an additional 30 min. Coverslips were washed as before and mounted onto glass slides using Prolong gold antifade reagent with DAPI (Invitrogen). Images were captured using an Olympus IX83 fluorescence microscope (Olympus, Richmond Hill, ON, Canada). 

### 2.6. Cell Proliferation 

A resazurin assay was used to measure cell viability/proliferation. Cells were seeded into 96 well plates at 2.0 × 10^3^ cells per well and incubated under normal culture conditions (complete medium, 37 °C, 5% CO_2_) for 48 h. Then resazurin (R&D Systems, Minneapolis, MN, USA) was added to each well according to the manufacturer’s instructions. After 4 h of incubation at 37 °C, fluorescence intensity was read at 544 nm excitation and 590 nm emission using a Varioskan LUX plate reader (Thermo Fisher). 

### 2.7. Transwell Migration and Invasion Assay 

Cell migration and invasion were assessed using cell culture inserts (Corning, Corning, NY, USA) according to the manufacturer’s instructions. Twenty-four-well transwell plates and inserts with 8 μM pores (Falcon, Corning, NY, USA) were used for these assays. For the migration assay, cells were serum-starved for 24 h, and 2.0 × 10^4^ cells (in 200 μL medium) were seeded in the upper chamber in serum-free media. A total of 600 μL of culture medium containing 10% FBS was added into the lower chamber. Cells were incubated at 37 °C for 24 h. Cells that had adhered to the upper surface of the insert were then removed using a cotton swab, and cells on the lower surface were washed with PBS, fixed with methanol, and stained with 1% crystal violet (BD Biosciences, Mississauga, ON, Canada) for 10 min at room temperature. The bottom of the insert was imaged using an inverted microscope at 100× magnification and a Moticam 10.0 MP camera (Motic, Richmond, BC, Canada). The number of cells in five fields, at 12, 3, 6, and 9 o’clock positions, as well as the center of each insert, was counted, and the average number of cells per field was calculated. For the invasion assay, the inserts were precoated with Matrigel Basement Membrane Matrix (300 µg/mL, Corning) according to the manufacturer’s instructions, and cells (2.0 × 10^4^ cells) were seeded in the upper chamber in serum-free media. A culture medium containing 10% FBS was added to the lower chamber. Cells were incubated at 37 °C for 48 h, then fixed, stained, and imaged with the same protocol as previously stated for the migration assay. 

### 2.8. Wound-Healing Assay 

Cells were seeded in 6-well plates (5.0 × 10^5^ cells/well) and allowed to grow to a confluent monolayer. Cell monolayers were wounded by scratching the well with a 200 μL pipette tip. The cells were washed three times with PBS and incubated at 37 °C until the wounds were closed. Wound closure was monitored over a series of time points by taking pictures using a light microscope with a Moticam 10.0 MP camera (Motic, Richmond, BC, Canada). The percentage of wound closure at each time point was calculated relative to the initial wound width.

### 2.9. Drug Sensitivity

Cells were seeded at 4.0 × 10^3^ cells per well in a 96-well plate and incubated for 24 h at 37 °C. After 24 h, the media was removed, and cells were treated in triplicate with increasing concentrations of cisplatin (Selleckchem, Houston, TX, USA) diluted in a complete culture medium. The control wells were treated with culture media and an equivalent volume of PBS. The cells were incubated with various concentrations of cisplatin for 48 h, and cell viability was assessed using the resazurin assay. Cell viability was calculated as relative viability at each concentration relative to that of the control group.

### 2.10. Colony Formation

Cells were plated into a 6-well plate at a concentration of 50 cells/well. Cells were grown for 7 days. Once colonies had formed, the cells were fixed with methanol and stained with 1% crystal violet. The colonies were counted and categorized into clustered, partially clustered, and spread colonies. 

### 2.11. Animal Studies 

All animal experiments were reviewed and approved by the University of Saskatchewan Animal Research Ethics Board (AREB). Male NOD.Cg-Prkdcscid Il2rgtm1Wjl/SzJ (NOD–SCID) were obtained from a breeding colony at the Laboratory Animal Services Unit, University of Saskatchewan. Male and female pairs for breeding were from the Jackson Laboratory. Cells were injected subcutaneously in the flank region of 4–8-week-old mice in 100 μL PBS. Tumors were measured using a digital caliper twice per week, and tumor volume was calculated by the formula (a^2^ × b)/2, where a and b are the short and long tumor diameters, respectively. Mice were euthanized when tumors reached approximately 10 × 10 mm in size. 

### 2.12. Immunohistochemistry

Tumor tissue samples were fixed in 10% buffered formalin solution for 48 h, processed, paraffin-embedded, and sectioned at a 5 µm thickness using a microtome. The sections were mounted on Superfrost Plus slides, deparaffinized in xylene, and rehydrated through graded ethanol to water. Antigen retrieval was performed using Tris-EDTA (pH 9.0) in a microwave for 20 min. Sections were incubated with a protein block for 15 min at room temperature. The primary antibody against EphA2 (#12927, Cell Signaling Technologies) was diluted in PBS containing 1% BSA and 0.3% Triton X-100 and added to sections, followed by incubation with the antibody for 1 h at room temperature. After three PBS washes, sections were incubated with HRP-conjugated secondary antibodies (Mouse specific HRP/DAB (ABC) Detection IHC Kit, Abcam, Toronto, ON, Canada, ab64259) for 30 min at room temperature. Visualization was achieved using the DAB Substrate Kit according to the manufacturer’s instructions, with counterstaining performed using hematoxylin where necessary. Imaging was performed using an Olympus BX41 light microscope (Olympus, Richmond Hill, ON, Canada), and images were captured with a DP74 camera.

### 2.13. Statistical Analysis 

Data are presented as the mean  ±  standard deviation (SD). Data distribution was tested by the Shapiro–Wilk normality test. Statistical significance was determined using a two-sided Student’s t-test and Mann–Whitney U-test. Statistical significance was defined as *p* < 0.05. The calculations were performed using GraphPad Prism version 9.4.0 for Windows (GraphPad Software, San Diego, CA, USA). 

## 3. Results

### 3.1. EphA2 Is Overexpressed in Canine and Human Osteosarcoma

**Figure 1:**
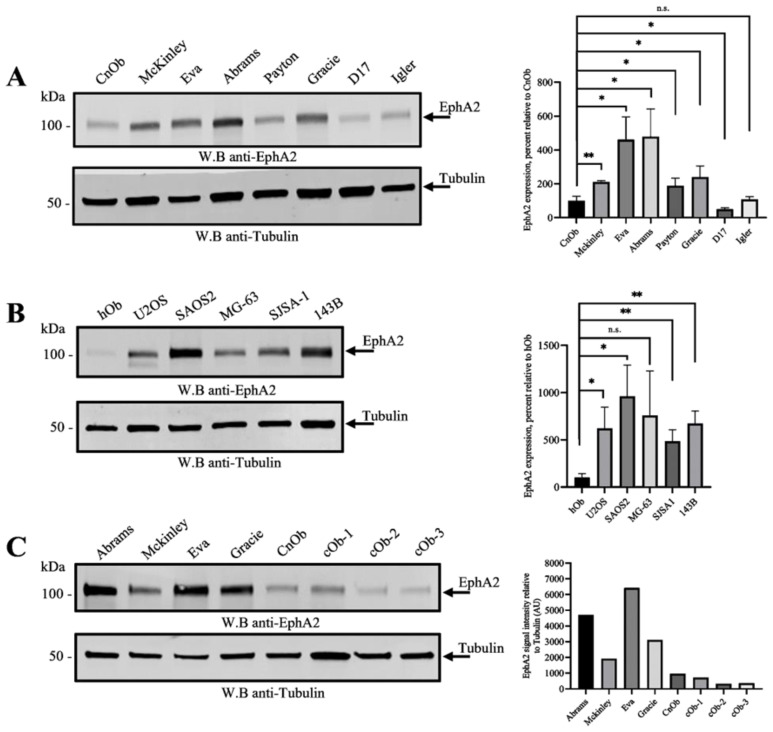
EphA2 is overexpressed in osteosarcoma cells. (**A**) Canine osteosarcoma cells Abrams, Eva, McKinley, Gracie, D17, Payton, and Igler, as well as non-malignant canine osteoblast cells (CnOb), were lysed and EphA2 expression was analyzed by Western blotting using an anti-EphA2 antibody. Tubulin expression was used as a loading control. EphA2 expression was quantified by densitometry, normalized on corresponding tubulin controls, and presented as percentages relative to osteoblast control (100%). The graph summarizes three independent experiments. (**B**) EphA2 expression in human osteosarcoma cells U2OS, SAOS2, MG-63, SJSA1, and 143B, as well as in non-malignant human osteoblast cells (hOb), was analyzed as in A. The graph summarizes three independent experiments. (**C**) EphA2 expression in the indicated canine osteosarcoma cell lines, a commercially available canine osteoblast cell line (CnOb), and 3 in-house preparations of canine primary osteoblasts (cOb-1-3) isolated from femoral bone samples were assessed by Western blotting and quantified as in A. The graph presents EphA2 signal intensity as arbitrary units (AU). * *p* < 0.05, ** *p* < 0.01; n.s. statistically not significant.

The EphA2 receptor has been associated with the progression of various types of human cancers [9,11]. To understand EphA2 expression in human and canine OS, we used Western blotting to analyze EphA2 expression levels in canine and human OS cell lines. EphA2 expression in five human (SAOS-2, U2OS, SJSA1, MG-63, and 143B) and seven canine (Abrams, Eva, McKinley, Gracie, D17, Payton, and Igler) OS lines was assessed and compared to that of normal human (hOb) and canine (CnOb) osteoblast cells (Figure 1A,B). The abundance of this protein in each cell line was evaluated by densitometry and normalized on a matching tubulin control. Our results demonstrated an increase in EphA2 levels in five human and five canine OS cell lines when compared to the canine and human osteoblast cells, respectively (Figure 1A,B; Supplementary Figures S1 and S2). To further confirm these findings, we assessed EphA2 expression in three additional canine primary osteoblast cell populations (cOb-1, 2, and 3). A low level of EphA2 expression was similarly found in these canine osteoblast cells when compared to OS cells (Figure 1C). EphA2 expression was also assessed in four canine OS tumor samples by immunohistochemistry. While EphA2 was expressed in tumor tissue, it was not detectable in osteoblast cells (Supplementary Figure S3).

### 3.2. EphA2 Enhances Viability of OS Cells

**Figure 2:**
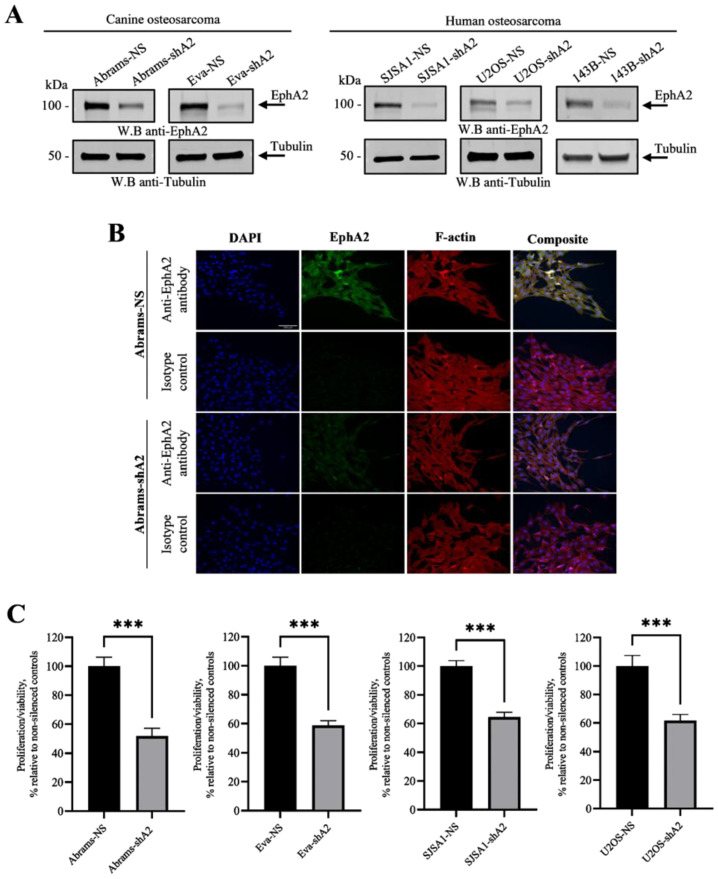
EphA2 promotes osteosarcoma cell proliferation. (**A**) Indicates that canine and human osteosarcoma cells were transduced with a specific shRNA targeting EphA2 (shA2) or with a non-silencing, scrambled shRNA (NS, control). EphA2 expression was then assessed by Western blotting using a specific anti-EphA2 antibody. (**B**) Abrams-NS and Abrams-shA2 cells were grown on glass coverslips to 70% confluency, fixed with 4% paraformaldehyde, and stained with anti-EphA2 (green), rhodamine phalloidin (cytoskeleton, red) and DAPI (cell nucleus, blue). Staining with a matching non-specific IgG was shown as specificity control. Fluorescent images were taken using an Olympus IX83 microscope at 200× magnification. Scale bar, 100 µm. (**C**) Indicates that cells were seeded in 96-well plates (2 × 10^3^ cells per well, *n* ≥ 6) and cultured for 48 h. To assess cell viability, resazurin was added to the wells, and the fluorescence was measured after 4 h using a Varioskan LUX plate reader. Data are shown as mean ± SD. Experiments were repeated three times. *** *p* < 0.001.

The EphA2 receptor has been demonstrated to enhance cell viability and survival in various human cancer types [15,16,17,18]. To evaluate any potential association between EphA2 upregulation in the OS lines and OS cell viability and survival, we silenced EphA2 expression in several human (SJSA-1, U2OS, and 143B) and canine (Abrams and Eva) OS lines (shA2; Figure 2A,B) exhibiting high endogenous EphA2 levels and examined their viability using the resazurin assay. Analyses of the resazurin assay revealed that EphA2 silencing significantly reduced the viability of both human and canine osteosarcoma cells when compared to matching non-silenced controls (Figure 2C).

### 3.3. EphA2 Supports OS Cell Migration and Invasion

**Figure 3:**
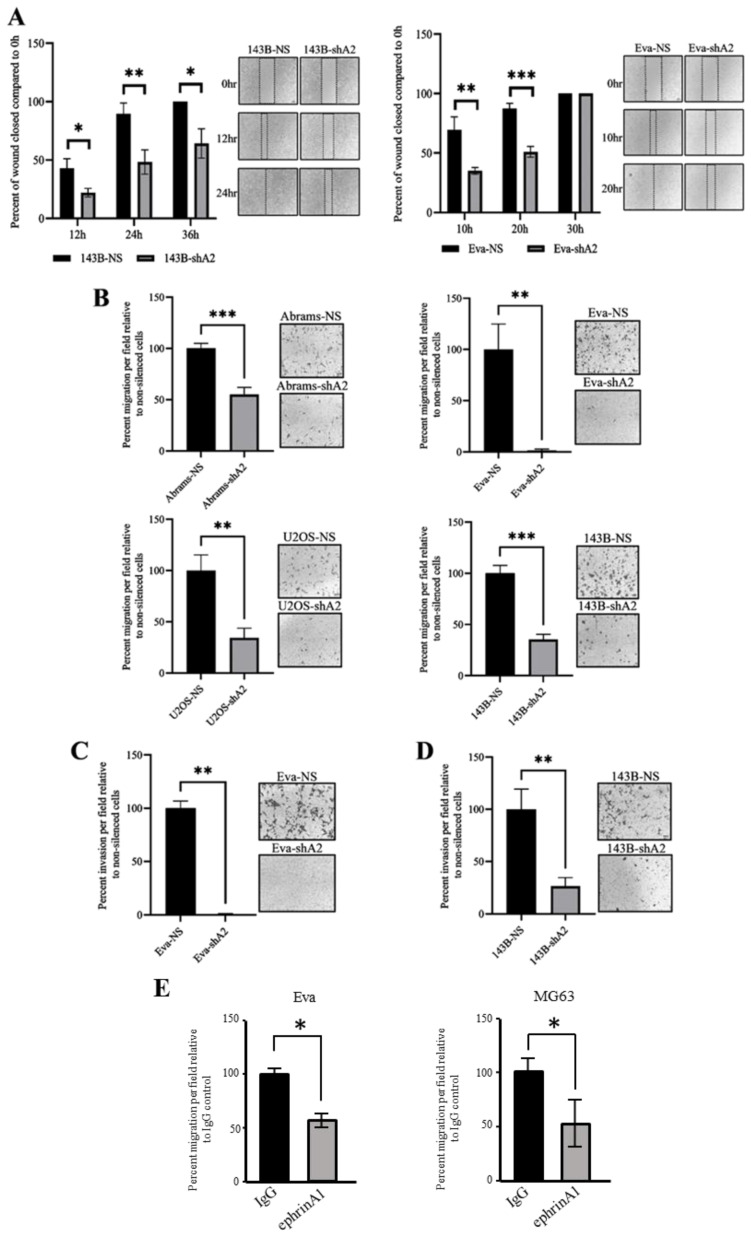
EphA2 affects osteosarcoma cell motility and invasion. (**A**) Human 143B-NS and 143B-shA2, and canine Eva-NS and Eva-shA2 cells were seeded into 6-well plates (5 × 10^5^ cells per well) in triplicates and cultured to form confluent monolayers. Using a 200 µL pipette tip, a wound/scratch was made in each monolayer, and wells were rinsed with medium to remove cell debris. Wound closure was monitored over time by imaging the same area of each wound (2 per scratch) using an inverted microscope at 40× magnification. Wound width in each image was measured using Microsoft PowerPoint. Graphs represent percentages of wounds closed at each timepoint relative to wound width at 0 h. Scale bar, 100 µm. (**B**) The indicated canine (**top**) and human (**bottom**) cells were seeded into transwell inserts (2 × 10^4^ cells per insert) in serum-free media (*n* = 3 per group). Cell culture media with 10% FBS was added to the lower chamber of each insert, and cells were incubated at 37 °C for 24 h. For assessing cell migration after 24 h, cells residing on the upper side of each insert porous membrane were removed by a cotton swab, and migrated cells at the lower surface of the membrane were fixed with methanol, stained with crystal violet, and counted. For cell counting, five selected fields of each insert were imaged at 100× magnification, and the average number of cells per field was calculated. Graphs represent percentages of migrated EphA2-silenced cells relative to that of matching non-silenced controls. (**C**,**D**) The indicated cells were serum-starved for 24 h and seeded into Matrigel-coated transwell inserts (2 × 10^4^ cells per well) in serum-free media (*n* = 3 per group). The lower chamber contained media with 10% FBS. After 48 h, the number of invaded cells at the lower surface of insert porous membranes were quantified and graphed as in (**B**). (**E**) Migration assays were conducted using canine Eva and human MG63 (3 × 10^4^ cells per insert) OS cells as in (**B**). Soluble ephrinA1 ligand or IgG (1 µg/mL) was added to the upper chamber at cell seeding. Graphs summarize data from two independent experiments and represent the percentage of migrated cells relative to IgG controls. Data are shown as mean ± SD. Experiments were repeated three times unless otherwise indicated. * *p* < 0.05, ** *p* < 0.01, *** *p* < 0.001.

For osteosarcoma patients, metastasis to the lung is particularly common and decreases patient survival [19]. To investigate the role of EphA2 in osteosarcoma cell migration, we used both wound healing and transwell migration assays. Our results indicate that silencing the EphA2 receptor significantly reduces the two-dimensional cell migration in both canine and human cell lines as assessed by the wound-healing assay (Figure 3A). We confirmed these findings using a transwell migration assay, where we observed a significant reduction in a targeted migration of canine and human EphA2-silenced OS cells, when compared to that of non-silenced controls (Figure 3B). Collectively, these results suggest that EphA2 supports migration of both canine and human osteosarcoma cells in culture. 

In addition to active migration, the invasion of cancer cells requires these cells to remodel the extracellular matrix and alter their cell–matrix and cell–cell contacts [20]. This is a pivotal characteristic of metastatic malignancies such as osteosarcoma [21]. To examine if EphA2 plays a part in regulating OS invasiveness, we utilized the Matrigel invasion assay. In line with our observations on cell migration, these experiments revealed that EphA2 silencing also reduces the ability of both canine and human OS cells to invade through Matrigel (Figure 3C,D).

To confirm if these effects were mediated by EphA2 silencing, we conducted transwell migration assays using canine Eva and human MG63 OS cells and assessed cell migration in the presence of the soluble EphA2 ligand, ephrinA1, or an IgG control. Pro-oncogenic effects of EphA2 in cancer cells are mainly orchestrated through ligand-independent activation of EphA2, and the presence of exogenous ephrinA1 induces EphA2 internalization and degradation and reduces its oncogenic signaling [22]. Indeed, transwell migration of both canine and human OS cells was significantly reduced in the presence of soluble ephrinA1 (Figure 3E).

### 3.4. EphA2 Enhances Resistance of OS Cells to Cisplatin

**Figure 4:**
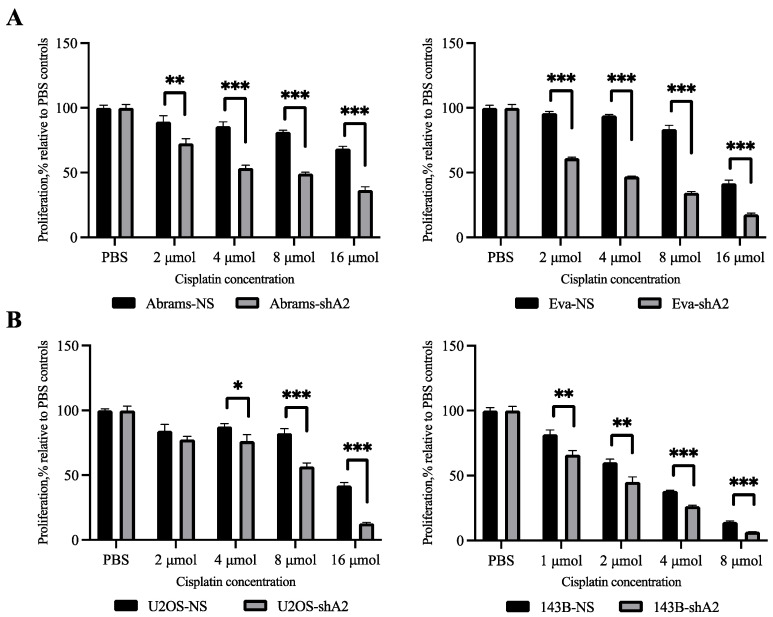
EphA2 increases resistance to cisplatin. (**A**) The indicated canine OS cells were seeded into 96-well plates (4 × 10^3^ cells per well) and allowed to adhere to the wells overnight. Culture media was replaced the next day with media containing increasing concentrations of cisplatin or media with PBS (as solvent control) at the volume matching the highest cisplatin dose as indicated (*n* = 3 per group). The treated cells were cultured for 48 h. To assess cell survival, resazurin was added to the wells, and the fluorescence signal was quantified as in Figure 2C. (**B**) The indicated human OS cells were tested for cisplatin sensitivity as described in A. Graphs represent cell survival as percentages relative to matching solvent controls. Data are shown as mean ± SD. Experiments were repeated three times. * *p* < 0.05, ** *p* < 0.01, *** *p* < 0.001.

Reduced sensitivity or resistance to chemotherapy is usually observed in invasive malignancies, including OS [23]. Increasing evidence also suggests that upregulation of EphA2 is associated with drug resistance in many human cancer types [24,25,26,27]. In OS, cisplatin is used as a chemotherapeutic drug; however, it is common for cisplatin resistance to be present or develop over time. To determine whether EphA2 contributes to the reduced sensitivity to cisplatin, EphA2-silenced and non-silenced OS cells were subjected to increasing doses of cisplatin, and their viability was assessed using the resazurin assay. These experiments showed that osteosarcoma cells were more sensitive to cisplatin after EphA2 silencing when compared to the non-silenced controls (Figure 4). Interestingly, this effect was consistently observed in both human and canine osteosarcoma cells (Figure 4A,B). This indicates that EphA2 contributes to reduced sensitivity to cisplatin in osteosarcoma and, therefore, may contribute to the development of drug resistance in this disease.

### 3.5. EphA2 Supports a More Invasive Phenotype in OS Cells

**Figure 5:**
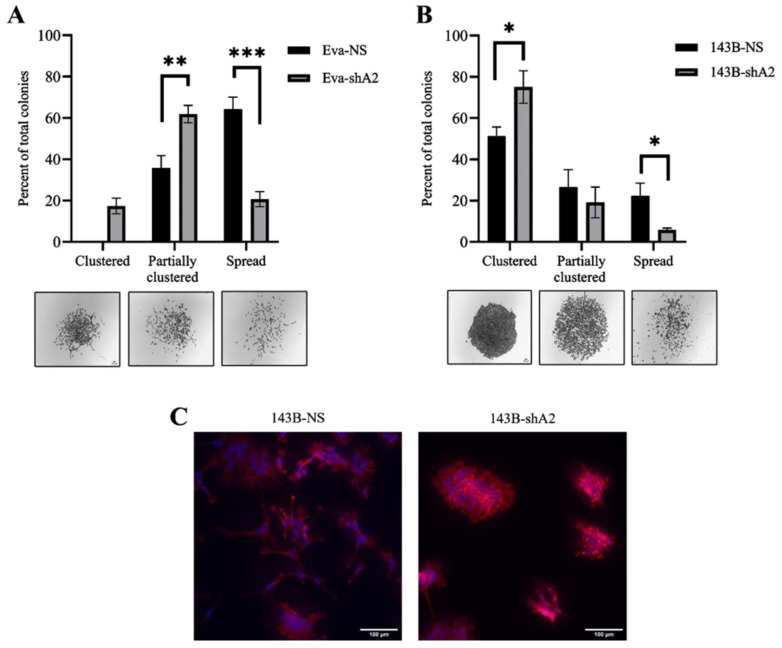
EphA2 affects osteosarcoma cell morphology. (**A**,**B**) The indicated canine and human OS cells were seeded into 6-well plates (50 cells per well, *n* = 3 per group) and allowed to grow into colonies for 7 days. Colonies were fixed with methanol and stained with crystal violet. The number of clustered, partially clustered, and spread colonies in each well were counted, and representative images were captured using a light microscope at 40× magnification. Graphs represent the proportion of each category of colonies relative to the total number of colonies. Representative images of these colonies are shown. (**C**) 143B-NS and 143B-shA2 cells were grown on glass coverslips to 50% confluency, fixed with 4% paraformaldehyde, and stained with rhodamine phalloidin (actin cytoskeleton, red) and DAPI (cell nucleus, blue). Fluorescent images were taken using an Olympus IX83 microscope at 200× magnification. Scale bar, 100 µm. Data are shown as mean ± SD. Experiments were repeated three times. * *p* < 0.05, ** *p* < 0.01, *** *p* < 0.001.

The EphA2 effects in supporting OS cell migration and invasion suggest a potential role for this molecule in regulating cell morphology. To evaluate EphA2’s influence on OS cell morphology, we used a colony formation assay. In this assay, EphA2-silenced and non-silenced cells were seeded to grow individual colonies, and then the morphological characteristics of these colonies were analyzed. In line with our findings supporting EphA2 roles in cell migration and invasion, silencing of the EphA2 receptor reduced the frequency of scattered colonies and enhanced the formation of compact colonies (Figure 5). This effect was more pronounced in the Eva canine OS cell line, since silencing of EphA2 shifted most Eva colonies from a spread morphology, with minimal cell–cell contacts, to a clustered or partially clustered morphology (Figure 5A). While a similar effect was observed in the 143B human OS cells (Figure 5B), EphA2 silencing also favored a rounder cell morphology compared to the non-silenced cells which have a more stellate shape (Figure 5C). Other studies have found that these differences in morphology, specifically round versus stellate, are correlated with less and more invasive behavior, respectively [28].

### 3.6. EphA2 Supports OS Tumor Development in Xenograft Models

**Figure 6:**
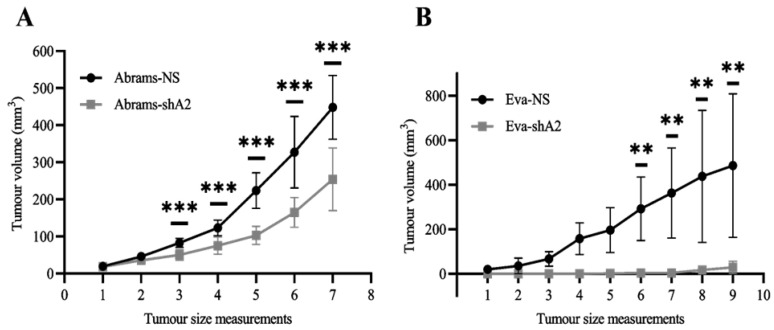
EphA2 promotes osteosarcoma tumor growth. (**A**) Abrams-NS and Abrams-shA2 osteosarcoma cells were injected subcutaneously in the flank area of 4–8-week-old male immunodeficient NOD–SCID gamma mice (1.0 × 10^6^ cells per mouse in 100 µL PBS, *n* = 4 per group). Tumor growth was monitored every 3–4 days, and tumor volume was calculated as a^2^ × b/2, where a and b are the short and long diameters, respectively. The graph represents a summary of two independent experiments. (**B**) Eva-NS and Eva-shA2 cells were injected and tumor growth was monitored as in A (*n* = 6 per group). The graph represents one of two independent experiments. Data are shown as mean ± SD. ** *p* < 0.01, *** *p* < 0.001.

Our findings indicate that EphA2 silencing decreases the viability, migration, and invasion of OS cells. Put together, these results suggest that EphA2 may also enhance the progression of OS tumors. To test this, we used mouse xenograft models. EphA2-silenced and non-silenced canine OS cells were injected subcutaneously in the flank region of NOD–SCID mice (1.0 × 10^6^ cells per animal), and tumor formation was monitored. We found that silencing EphA2 significantly decreased the growth of tumors formed by Abrams OS cells (Figure 6A). Interestingly, this effect was even more pronounced in Eva xenografts, as both tumor initiation and growth were suppressed by EphA2 silencing (Figure 6B). Overall, these results strongly suggest that EphA2 activity is essential for OS development and progression.

### 3.7. EphA2 Is Associated with the Activation of Various Signaling Pathways

To gain insight into the cytoplasmic signaling mechanisms utilized by EphA2, we assessed the activation status of multiple signaling pathways in both human and canine osteosarcoma cells. EphA2 silencing was consistently associated with reduced activating phosphorylation of AKT, ERK1/ERK2, and SRC kinases in both human and canine osteosarcoma cells. However, reduced activating phosphorylation of mTOR was only evident in canine Eva cells. Additionally, while reduced expression of integrin β3 was observed in both human and canine osteosarcoma cells following EphA2 silencing, a reduction in N-cadherin expression was only observed in canine Eva cells.

## 4. Discussion

Osteosarcoma is a highly malignant and aggressive cancer that constitutes most primary bone tumors in humans and dogs [1,29,30]. Despite a poor prognosis, treatment options for OS have remained unchanged for decades, highlighting the imperative need for new therapeutic avenues. Employing a comparative oncology approach, we have demonstrated the upregulation of the EphA2 receptor in OS cells, correlating with the aggressive behavior of both human and canine OS cells. Our data specifically indicate that EphA2 enhances the viability, migration, and invasion of OS cells in culture and promotes OS xenograft tumors. While the overexpression of EphA2 in human Ewing sarcoma (ES) and OS tumor samples [31,32,33] and cell lines [33] has been reported previously, our study represents the first report of EphA2 expression in canine OS cells and tumors. To explore whether this receptor plays similar roles in both species, we silenced its expression in multiple human and canine OS cell lines. We reported the alterations in OS cell functions and assessed the activating phosphorylation status of cell signaling pathways in a comparative manner to underscore the similarities in this disease between humans and dogs. These parallels in the expression and functional roles of the EphA2 receptor in both species support the rationale for targeting EphA2 to suppress OS in both humans and dogs. Furthermore, considering dogs’ shorter lifespan and higher rate of OS development compared to humans, studying OS in spontaneous canine models can expedite clinical trials of potential therapeutics, ultimately generating improved treatment options for both species [4].

**Figure 7:**
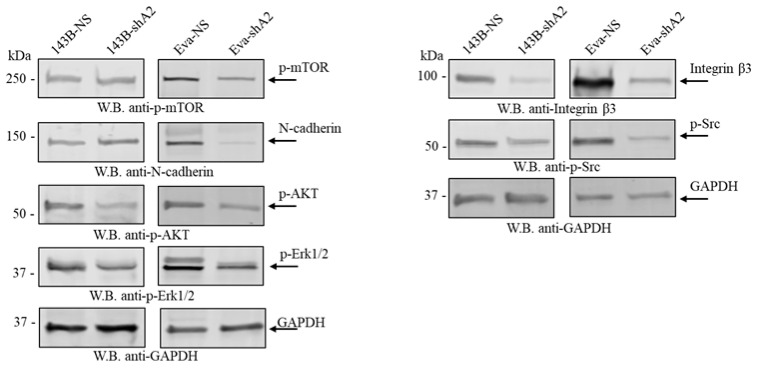
EphA2 affects multiple signaling pathways in human and canine osteosarcoma cells. The indicated cells were cultured to approximately 70% confluency, serum-starved overnight, and the phosphorylation status of various proteins, as well as the expression of N-cadherin and Integrin β3, was assessed by Western blotting.

Enhanced cell proliferation and reduced apoptosis are critical characteristics of cancer cells that promote their viability and survival [34]. Multiple Eph receptor tyrosine kinases, particularly EphA2, are overexpressed in various cancers and mediate pro-oncogenic signaling mechanisms in cancer cells [9,35]. Although the specific signaling mechanism of how EphA2 regulates responses in OS cells is unknown, previous reports in other cancer types highlight a ligand-independent pro-oncogenic mode of action of the EphA2 receptor [36]. Specifically, it was found that phosphorylation at residue S897 within the EphA2 intracellular domain leads to the activation of downstream signaling mechanisms in cancer cells without requiring a ligand–receptor interaction [26,35]. These signaling mechanisms include AKT-mTORC1, Raf-MEK-ERK, SRC-ERK, and RhoG-PI3K/AKT, which, upon activation, enhance cell proliferation, survival, and drug resistance [35,37,38]. Interestingly, emerging evidence suggests that phosphorylation of EphA2 at S897 is also regulated by AGC protein kinases, RSK, and PI3K/AKT, depending on the stimuli and cell types [35,38,39,40]. In line with these reports, we observed a reduced activating phosphorylation status of SRC, AKT, and ERK1/2 in both human and canine osteosarcoma cells upon silencing of the EphA2 receptor expression (Figure 7); however, mTOR phosphorylation was affected only in canine OS cells, possibly reflecting some cell line variations.

Our experiments revealed that silencing EphA2 expression in both canine and human osteosarcoma cells makes them more sensitive to cisplatin. Cisplatin is a common chemotherapeutic agent frequently used for the treatment of both canine and human osteosarcoma patients [3,4]. Interestingly, Moyano-Galceran et al. showed that platinum-based chemotherapeutic drugs induce oxidative stress, which, in turn, triggers ERK1/2 and RSK activation [27]. Our original results showed that silencing EphA2 was associated with reduced activation of ERK1/2, SRC, and AKT, which are key pathways involved in cancer cell survival and proliferation. This suggests that EphA2 may support osteosarcoma cell survival by maintaining the activation of these pathways. Since activated ERK and RSK proteins can enhance the phosphorylation of EphA2 at S897, this could provide further support to cancer cell survival when EphA2 is present [27]. Therefore, the reduced activation of ERK1/2, SRC, and AKT upon EphA2 silencing could explain the increased sensitivity to cisplatin, as these cells are less able to counteract the drug-induced oxidative stress. In line with our observations in OS, increased sensitivity of cancer cells to cisplatin associated with EphA2 inhibition was previously observed in human gastric [41], lung [42], and esophageal [43] cancer models. Taken together, EphA2 enhances cell viability and survival in both human and canine OS cells. Cisplatin is one of the most commonly used chemotherapeutic drugs; however, its application is associated with major toxic side effects [44]. Combining cisplatin with other approaches, such as EphA2 inhibition, could potentially reduce these side effects and improve patient outcomes. However, further studies are required to explore the potential implications of combining chemotherapy and EphA2 inhibition in OS treatment.

Regarding our findings that the EphA2 receptor promotes migration and invasion of OS cells, a previous investigation of the role of EphA2 in Ewing sarcoma (ES) determined that high EphA2 expression is associated with the metastatic phenotype. In this work, the migratory and invasive abilities of human ES cells were found to be decreased when the EphA2 expression was silenced [45]. This effect was also observed in some other human malignancies, such as colorectal cancer [46], glioma [38], and non-small cell lung carcinoma [47]. Here, we observed that EphA2 silencing reduced the expression of integrin β3, a well-known mediator of cell adhesion and migration [48,49], in both human and canine OS cells (Figure 7). The interaction between integrins and the extracellular matrix facilitates signaling pathways that promote cytoskeletal rearrangements and cell motility. Reduced integrin β3 expression upon EphA2 silencing likely disrupts these interactions, impairing the cells’ ability to migrate and invade surrounding tissues, as reported here. This finding aligns with previous reports highlighting the role of integrin β3 in enhancing human osteosarcoma cell migration and invasion [49,50]. Furthermore, the observed reduction in integrin β3 expression upon EphA2 silencing may also be related to downstream effects on the AKT and ERK–MAPK pathways. These pathways are crucial for integrin signaling and cell migration, and their reduced activation, as seen in our study, can lead to decreased integrin-mediated cell adhesion and motility. Thus, our results underscore the importance of EphA2 in promoting osteosarcoma invasiveness, at least in part through the regulation of integrin β3 and associated signaling pathways. Although not investigated here, it is plausible that EphA2 is able to operate by activating cytoskeleton regulators, including RhoG [51]. Kawai et al. reported that when EphA2 is activated in a ligand-independent manner via phosphorylation at the S897 residue, it interacts with Ephexin 4, and this triggers the activation of the RhoG GTPase [51]. Active RhoG, through downstream molecules, activates the GTPase Rac, which in most cells initiates leading protrusions, one of the first key steps in cell migration and invasion [20,51,52].

In addition to observing the effects of EphA2 silencing in cultured human and canine osteosarcoma cells, we also found that suppressing EphA2 activity significantly decreases tumor growth in xenograft models of canine osteosarcoma. These observations agree with the results of similar studies done in human cervical cancer [53] and triple-negative breast cancer [16], where EphA2 knockdown significantly decreased tumor growth in vivo. 

Collectively, our data suggest that targeting EphA2, either alone or in combination with chemotherapy, holds promise for improving outcomes in osteosarcoma patients. Given the active pursuit of combination therapies to overcome resistance and reduce side effects, combining cisplatin with anti-EphA2 therapies emerges as a potential avenue for enhancing treatment efficacy. Notably, the anti-EphA2 monoclonal antibody DS-8895a has demonstrated encouraging results in recent studies. In breast and gastric cancer xenograft models, DS-8895a significantly decreased tumor growth, and its combination with cisplatin exhibited a more pronounced reduction in tumor development compared to individual therapies [41]. Progressing to clinical trials, phase 1 trials confirmed the safety and tolerability of DS-8895a in patients [54,55]. The targeting of EphA2 proves advantageous, given its association with the activation of key oncogenic signaling pathways and invasive traits. Our observations suggest that extending the targeting of EphA2 to human and canine osteosarcoma may reduce cells’ ability to evade elimination by cisplatin treatment while concurrently suppressing their invasive potential.

## 5. Conclusions

Comparative oncology is an emerging field with the goal of advancing research on cancers that exhibit striking similarities between canines and humans. In this study, we utilized human and canine osteosarcoma cell lines in side-by-side comparisons and confirmed the involvement of the EphA2 receptor in the malignant progression of osteosarcoma in both species. These observations emphasize the translational potential of studying cancers in canines and applying the gained knowledge to human malignancies. Together, our findings point toward the EphA2 receptor as a promising target for treating osteosarcoma in both human and canine patients.

## Data Availability

The original contributions presented in the study are included in the article/Supplementary Material. Further inquiries can be directed to the corresponding author.

## Supplementary Materials

The following supporting information can be downloaded at: https://www.mdpi.com/article/10.3390/cells13141201/s1, Figure S1: EphA2 expression in canine osteosarcoma and osteoblast cells; Figure S2: EphA2 expression in human osteosarcoma and osteoblast cells; Figure S3: EphA2 expression in canine osteosarcoma tumors.
