# Supplementary material for: The EphA2 Receptor Regulates Invasiveness and Drug Sensitivity in Canine and Human Osteosarcoma Cells
Source: Cells. 2024 Jul 16;13(14):1201. doi: 10.3390/cells13141201 (PMC11275032; doi:10.3390/cells13141201)
Supplement: Supplementary file 1 [file cells-13-01201-s001.zip › cells-2896724-supplementary.pdf]

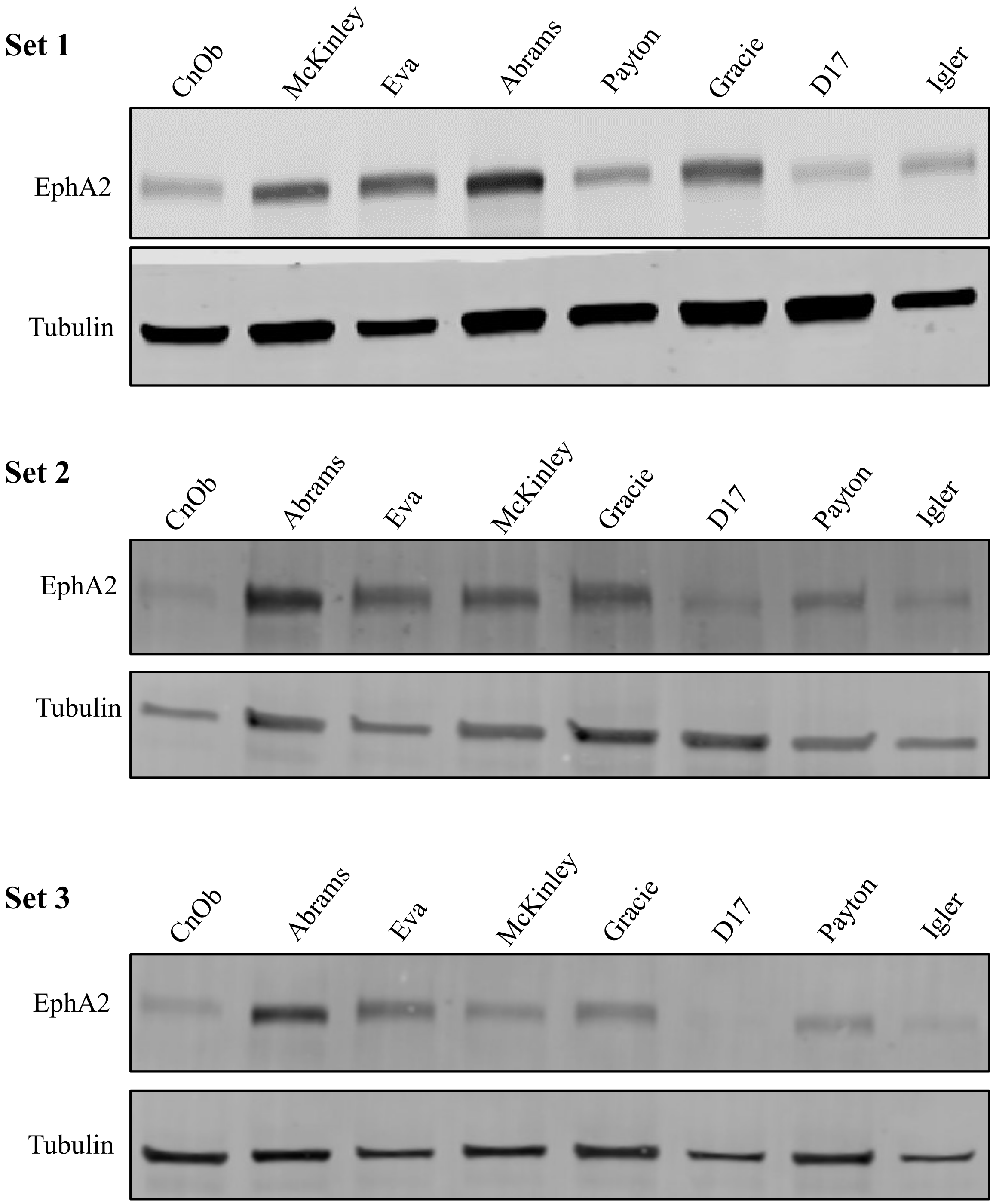


**Supplementary Figure S1**. EphA2 expression in canine osteosarcoma and osteoblast cells. Three independent sets of cell lysates were prepared and assessed by Western blotting.

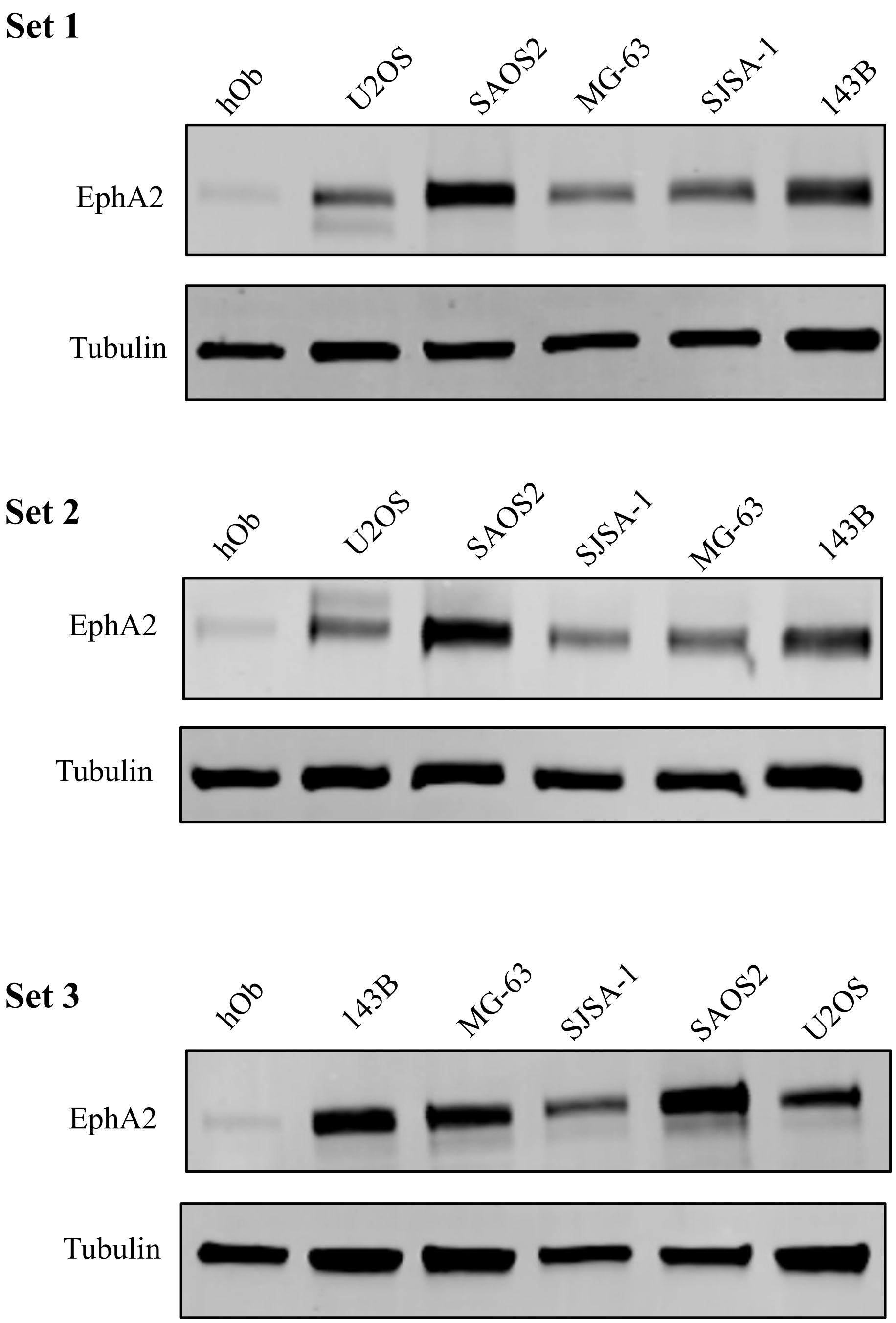


**Supplementary Figure S2**. EphA2 expression in human osteosarcoma and osteoblast cells. Three independent sets of cell lysates were prepared and assessed by Western blotting.

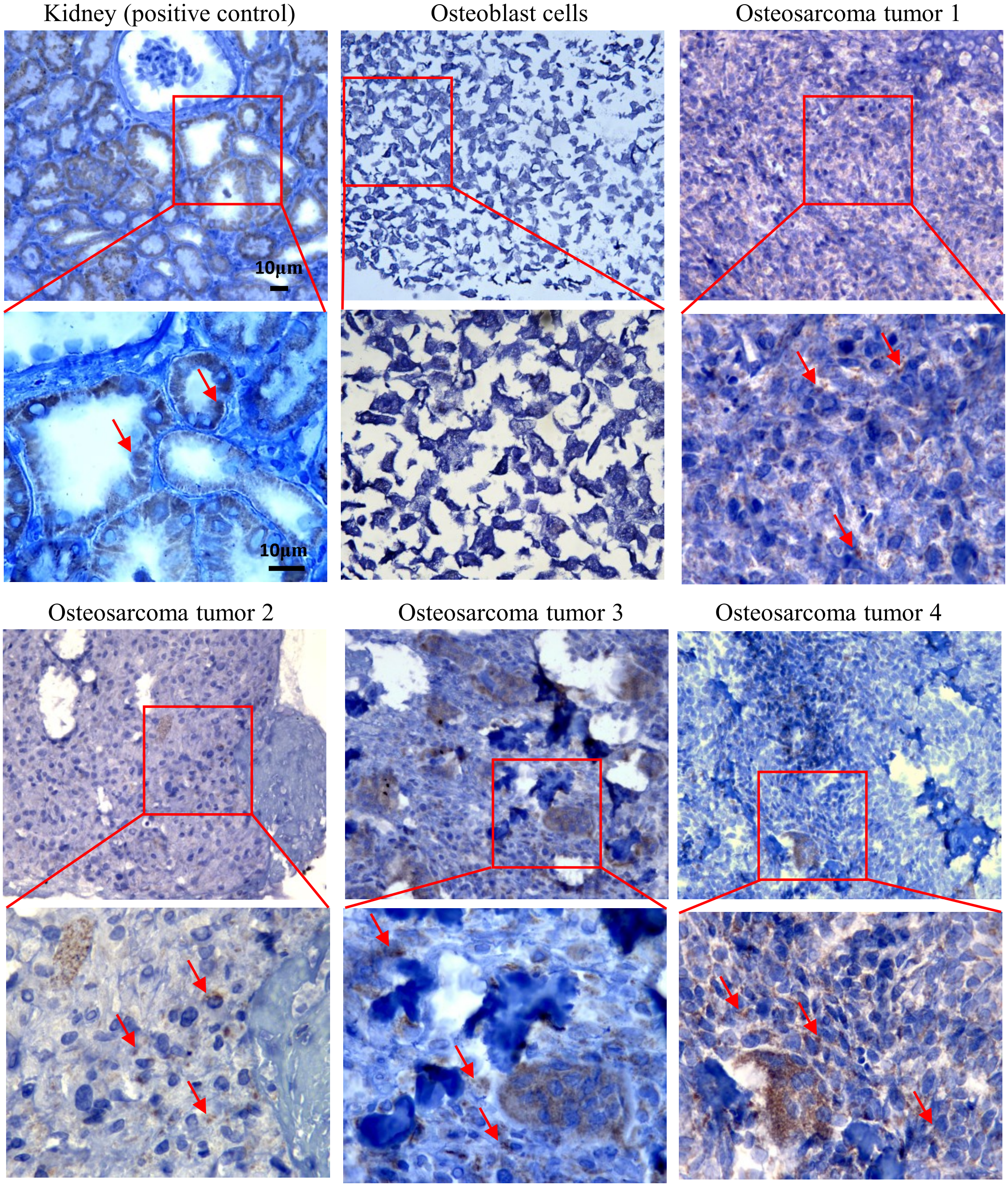


**Supplementary Figure S3**. EphA2 expression in canine kidney tissue, osteoblast cell pellet, and four osteosarcoma tumors was assessed by immunohistochemistry using a specific anti-EphA2 antibody. Canine osteoblast cells (CnOb) were cultured, collected, and pelleted by centrifugation. The osteoblast cell pellet was then fixed and prepared for paraffin embedding. Sections from these osteoblast cell blocks were used for immunohistochemical staining.
